# Recruitment of patients with Chronic Obstructive Pulmonary Disease (COPD) from the Clinical Practice Research Datalink (CPRD) for research

**DOI:** 10.1038/s41533-018-0089-3

**Published:** 2018-06-19

**Authors:** Jennifer K. Quint, Elisabeth Moore, Adam Lewis, Maimoona Hashmi, Kirin Sultana, Mark Wright, Liam Smeeth, Lia Chatzidiakou, Roderic Jones, Sean Beevers, Sefki Kolozali, Frank Kelly, Benjamin Barratt

**Affiliations:** 1 Department of Respiratory Epidemiology, Occupational Medicine & Public Health, Imperial College London, National Heart and Lung Institute, London, UK; 2 Clinical Practice Research Datalink, Medicines and Healthcare products Regulatory Agency, London, UK; 3 Department of Epidemiology & Population Health, London School of Hygiene & Tropical Medicine, London, UK; 4 Department of Chemistry, University of Cambridge, Cambridge, UK; 5 Analytical & Environmental Sciences Division, King’s College London, London, UK; 6 NIHR Health Protection Research Unit in Health Impacts of Environmental Hazards, King’s College London, London, UK

## Abstract

Databases of electronic health records (EHR) are not only a valuable source of data for health research but have also recently been used as a medium through which potential study participants can be screened, located and approached to take part in research. The aim was to assess whether it is feasible and practical to screen, locate and approach patients to take part in research through the Clinical Practice Research Datalink (CPRD). This is a cohort study in primary care. The CPRD anonymised EHR database was searched to screen patients with Chronic Obstructive Pulmonary Disease (COPD) to take part in a research study. The potential participants were contacted via their General Practitioner (GP) who confirmed their eligibility. Eighty two practices across Greater London were invited to the study. Twenty-six (31.7%) practices consented to participate resulting in a pre-screened list of 988 patients. Of these, 632 (63.7%) were confirmed as eligible following the GP review. Two hundred twenty seven (36%) response forms were received by the study team; 79 (34.8%) responded ‘yes’ (i.e., they wanted to be contacted by the research assistant for more information and to talk about enrolling in the study), and 148 (65.2%) declined participation. This study has shown that it is possible to use EHR databases such as CPRD to screen, locate and recruit participants for research. This method provides access to a cohort of patients while minimising input needed by GPs and allows researchers to examine healthcare usage and disease burden in more detail and in real-life settings.

## Chronic lung disease: Screening for patient participants

Screening anonymized electronic health records could prove a valuable, time-saving method for identifying patient cohorts for research projects. Jennifer Quint at Imperial College, London, and co-workers used primary care databases provided by doctors’ surgeries in London to find suitable patients for a study monitoring daily chronic obstructive pulmonary disease (COPD) symptoms. Using carefully-designed algorithms, the researchers identified 988 COPD patients who met eligibility criteria and lived within defined localities. Quint’s team then asked the patients’ doctors to review and approve the list for their own practice, thus limiting the doctors’ workload for selecting patients. The researchers approached 632 patients to invite them to participate in the research; 66 were enrolled. This provided an adequate number for the study, though the team highlight a need to improve strategies that encourage patients to take part in research.

## Introduction

Recruitment for health research traditionally involves approaches being made to individual healthcare professionals to undertake a time-consuming, labour intensive search of their own patient records. In an increasingly demanding clinical setting, this may limit opportunity for participant uptake.

More recently, studies have adopted other means, such as utilising electronic health records (EHR) to screen and locate potentially suitable participants nationally. This method has been demonstrated to be effective in a genotoxicity study requiring the collection of a biosample from patients^1^ and in a cluster randomised asthma study involving the delivery of a simple intervention.^2^

The present study investigated the association between environmental exposures and exacerbations of Chronic Obstructive Pulmonary Disease (COPD).^3^ The Clinical Practice Research Datalink (CPRD) anonymised General Practitioner (GP) record database was used to screen and locate eligible study participants, in a restricted geographical region, who were identified at the practice and invited to take part in the study. Participants were required to carry an environmental monitoring device for 6 months, undertake lung function tests and complete diary symptoms cards; response rates and lessons learned from using this method are examined in this report.

## Results

**Fig. 1:**
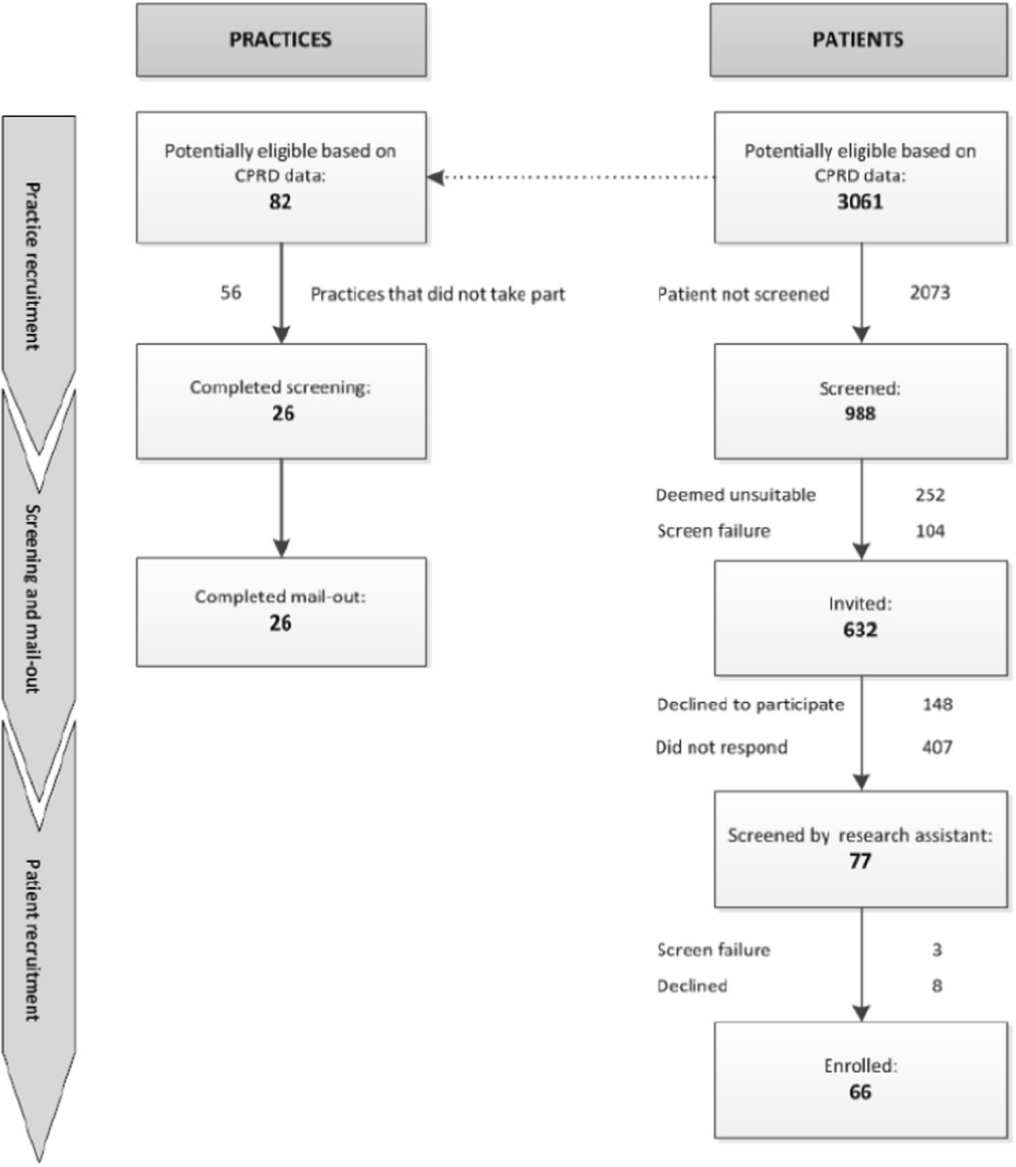
COPE study patient eligibility flow

Figure 1 shows the flow of potentially eligible patients and their practices through the study. The study stage is indicated by the column on the left and corresponding practice and patient numbers are shown. Eighty-two practices were invited to the study of which 56 (68.3%) did not take part, of these 17.9% indicated they had ‘too much workload at present’ to complete the study activities and a small number expressed reasons of ‘insufficient resources’ or ‘not enough remuneration’, this effectively resulted in a loss of 2073 (67.7%) patients who might otherwise have been considered in the study. Twenty-six (31.7%) practices consented to participate resulting in a pre-screened list of 988 patients. Of these, 632 (63.9%) were considered eligible following GP review and 359/988 (36.3%) excluded; 104 (10.5%) no longer fitted the study criteria (e.g., no diagnosis of COPD, no exacerbations in the last year, or patient was a current smoker), and a further of 255 (23%) deemed unsuitable to participate by the GP; 220 (22.3%) were either housebound, suffering from dementia or other co-morbidities, and 35 (3.5%) had transferred from the practice and were no longer contactable for the study.

The patient recruitment period ran from December 2015 to February 2017. GP practices were invited to take part with some practices being approached several times.

**Fig. 2:**
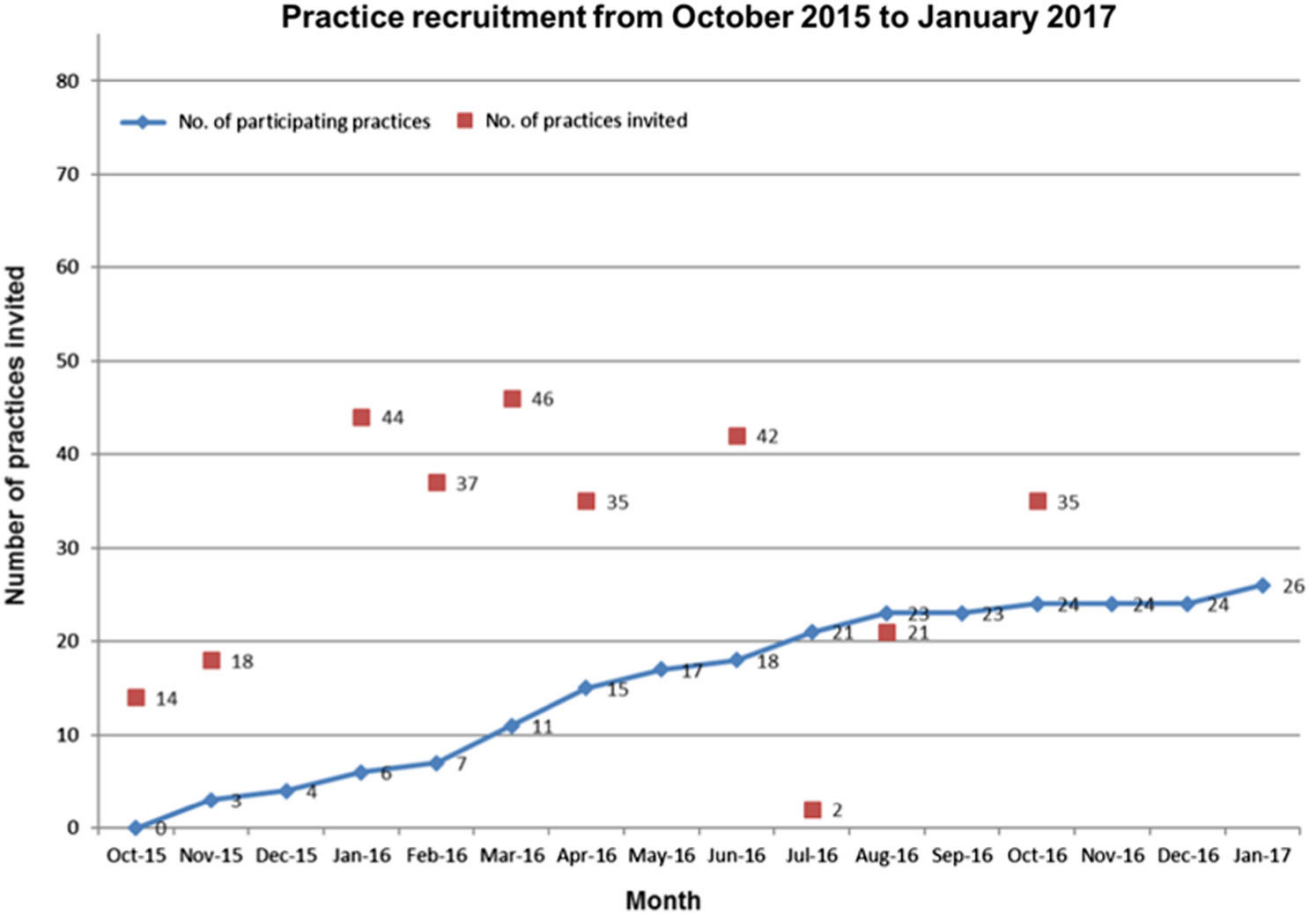
COPE site recruitment

Figure 2 shows the number of GP practices invited to participate during the recruitment phase and the number of participating practices. Recruitment was slower during the winter period. Searches were adapted (e.g., a wider area searched) during the course of the recruitment period.

**Fig. 3:**
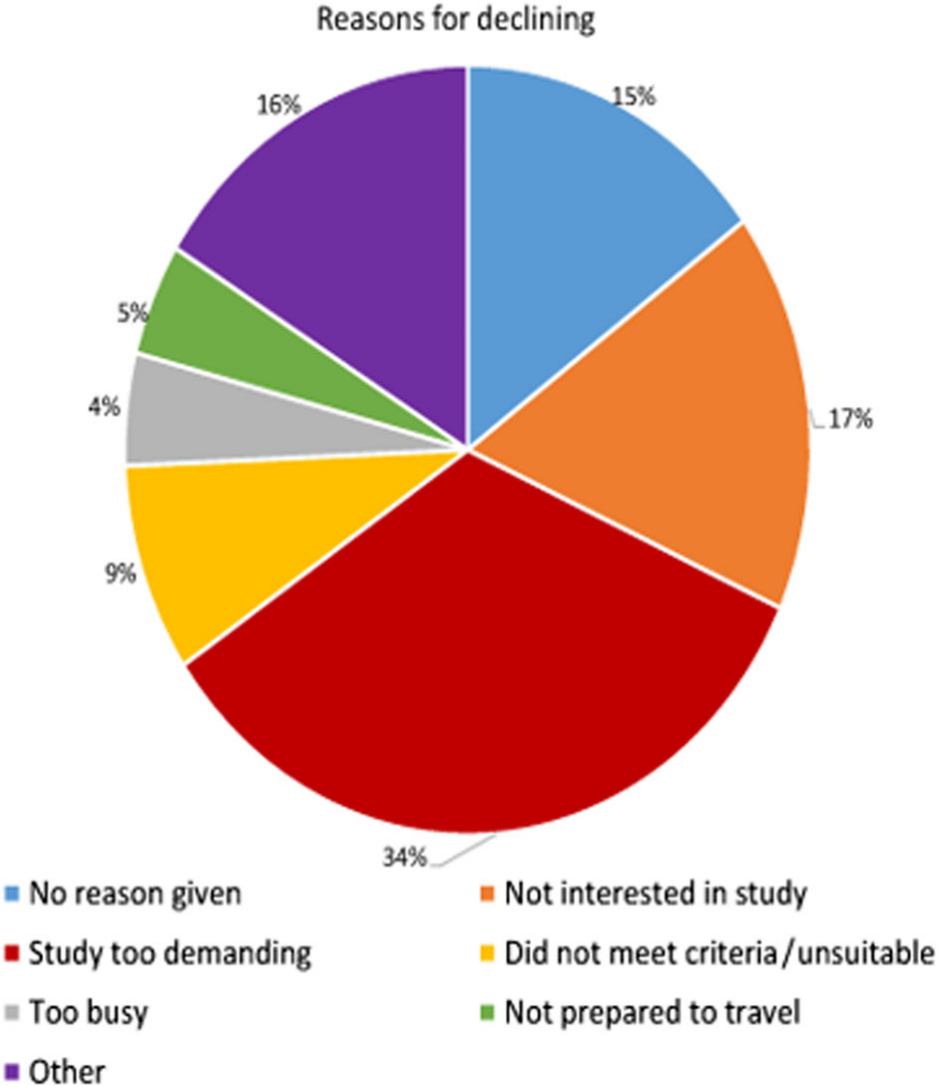
Reasons for declining to participate

A total of 632 patients were approached and returned 227 (36%) response forms: 148 (65.2%) declined participation, 79 (34.8%) responded ‘yes’ (i.e., they wanted to be contacted by the research assistant for more information and to talk about enrolling in the study) and 66 (29%) were enrolled. Figure 3 shows the main reasons for patients declining were ‘study too demanding’ (34%), and ‘not interested in the study’ (17%).

## Discussion

### Summary

This study has demonstrated how an EHR database can be used to screen suitable participants and locate their registered practice, minimising input needed by busy GPs and health care professionals in secondary care. This strategy reduces the recruitment burden and increases the recruitment pool. In addition, patients may be offered the chance to take part in research who otherwise may not be presented with this opportunity as they are not known to secondary care or do not frequently visit their GP.

### Strengths and limitations

One of the advantages of this method is that researchers can link EHR, with data on medications and GP visits, to hospital visits (Hospital Episode Statistics) and mortality records (Office of National Statistics) to examine healthcare usage and disease burden in more detail. This reduces information that needs to be self-reported by study participants thus shortening their study visits, and improving accuracy of data collected (sometimes exact timing of events cannot be recalled by patients, in particular exact timing of acute events^4^). Furthermore, the health data can be linked to other data, for example in the case of the COPE study to diverse exposure estimations (e.g., data from air quality monitors/mobility data).

This method relied on GPs reviewing a pre-screened patient list and sending out study information packs. Although GPs were reimbursed for taking part, some GP practices declined participation because of busy workloads, or did not respond resulting in almost two thirds of patients excluded from being screened for this study (Fig. 1). This approach is limited to GP practices registered with electronic healthcare systems such as CPRD but many are not and therefore there are patients who are not accessible through this method. 31.7% of eligible practices distributed across London agreed to participate in this study. Whilst CPRD invited practices in the greater London area to take part, there is no discernible difference between practices who agreed to take part or did not agree to suggest this would result in patient selection bias. The only difference between those practices that took part and those that didn’t was due to available workload at the time of being approached.

We used validated definitions with a high positive predictive value (PPV) to identify those with COPD and so it is unlikely that eligible patients will have been missed, however, we recognise that there are likely to be some coding differences between GPs but were not able to evaluate this misclassification. Despite using the validated codelists and algorithm, some patients no longer fitted the study criteria perhaps because their diagnosis had changed over time. Within EHRs one diagnosis may not supersede a previously recorded diagnosis which was included in the screening criteria. The majority of these were identified by the GP and a much smaller number subsequently by the research team. This is unlikely to be a COPD specific phenomenon and needs to be considered when using this method for any disease. This also highlights the importance of periodically reviewing the search criteria or presenting up to date pre-screened patient lists for GP review to reduce the possibilities of inviting patients who no longer meet the eligibility criteria. GP screening was also useful in indicating those patients that were unable/unsuitable to take part for reasons such as being housebound or who had co-morbidities that may limit participation. Often this was for social reasons or frailty, which would not have been readily detected from the study search algorithms alone or were not included in the search engine and suggests further improvements can be made to the search criteria. The final number of patients enrolled from CPRD into the study was 66 (41%) of the 160 patient recruitment target (6.6% patients enrolled from 988 screened patients); 227/629 (36%) patients contacted the research site of which 29% (66) were enrolled. A higher percentage of patients took part in the study than GPs despite the demands of the study on the patient.

Primary care recruitment numbers to trials using CPRD are likely to be higher than from practices who do not use CPRD as is shown in comparison to Davey et al.^5^ who achieved a recruitment rate of 2.3% by using similar methods without the collaboration of CPRD or a double screening process. In the COPE study, all GP practices and participants understood issues of access to patient records and confidentiality within CPRD contract agreements and Patient Study Information Sheets. The codelists and algorithms used have previously been validated and all GP practices and participants understood issues of access to patient records and confidentiality within CPRD contract agreements and Patient study Information Sheets. To explain in more detail; CPRD has a pseudo-anonymised database and only the GP can identify the patient. CPRD operates an opt-in model whereby a GP practice must provide consent for CPRD to collect de-identified primary care data from their practice. This consent must first be in place to enable extractions of data from the primary care EHR. Patients at participating practices have the right to opt-out of sharing and use of their data for research at any time and CPRD respects this choice. Patients are informed that their data are used for public health research and how they can opt-out, via a Fair Processing Notice displayed in the practice waiting room. The CPRD website also provides detailed information for the public informing them of the uses of their data for research purposes and the legal and ethical permissions obtained. GP practices who agreed to participate in this research did so with the knowledge that patient anonymity would be protected. No informed consent is necessary in order to receive information packs.

### Comparison with existing literature

Whilst data from EHRs have been used in thousands of peer reviewed papers, there has been less use of EHR for patient recruitment in studies. Those studies that have utilised EHR to screen for potentially suitable participants for recruitment have been in different disease areas including cardiovascular,^1^ asthma,^2^ and osteoarthritis.^5^ From the latter study there is an inference that this approach may not be optimal and other methods such as newspaper recruitment may be cheaper and easier. Recruitment in primary care is used in the COPE study to provision a study in secondary care. Importantly the findings of the COPE study demonstrates that EHR recruitment in primary care is a feasible approach that can be optimised.

We feel that this recruitment strategy may not be directly compared to strategies used by the pharmaceutical industry, although we acknowledge that a few pharmaceutical companies may use PIC as a strategy for recruitment of patients. There are significant differences in the amounts of funding available in research with Pharmaceutical company involvement. For example, the £80 million pound investment by GSK in the Salford Lung Study^14^. Significant pharmaceutical trials do not state their recruitment strategy within their methodology.^6–9^ Only one of these trials stated how many patients were screened^8^ and it is likely with the numbers of centres participating (356 centres in 43 countries) that screening was performed directly within secondary care, and therefore a highly specific group of patients. Furthermore, the concept and delivery of drug trials compared to our study are significantly different.

Patients received a personalised feedback report but were not offered any change or potential improvement to their direct medical care as part of our study and this may be a reason for a lower why recruitment rate than might be seen for a drug treatment study. Indeed a systematic review of recruitment strategies suggests that recruitment into trials is higher when patients are offered free medication.^10^ Ngune et al.^10^ also recommend providing financial incentives. In this study, participants were offered £20 for participation and GP practices were paid up to £100 for reviewing patient screening lists and £25 per patient enrolled to be involved in this study.

### Implications for research and clinical practice

This paper presents the findings of a novel recruitment approach for a study in secondary care based in primary care, and that may inform care of COPD patients in primary care. Thus providing access to a cohort of patients whilst minimising input needed by GPs and by researchers in secondary care. Furthermore, this study demonstrates that patients can be conveniently located in a geographical location for a secondary care site, reducing the research burden for patients. This recruitment methodology is not previously published in the BMJ Open protocol paper.^3^ This strategy is applicable to other regions in the UK, and to other specialities besides respiratory care. A further advantage of this method is that researchers can link data from a variety of sources such as electronic healthcare records, secondary data repository such as Hospital Episode Statistics (HES) together with data obtained directly from the patient (such as patient diaries and additional tests). An adequate number of patients can be enrolled by this method, as evidenced in this study, but thought needs to be given to increasing patient participation, for example looking at strategies to reduce research burden.

## Methods

CPRD holds an anonymised GP records database containing continually updated primary care medical data. This database includes details on symptoms, diagnoses, tests, prescriptions, patient demographics, health behaviours, and referrals to secondary care. Details in CPRD are mainly recorded using a system of Read codes, which is a polyhierarchical terminology system. The CPRD database is broadly representative of the UK general population.^11^

Validated codelists and algorithms^12,13^ created by members of the study team were used by CPRD researchers to screen patients based on the following eligibility criteria; diagnosis of moderate or severe COPD, with at least one COPD exacerbation in the year preceding study start, age 35 and above with evidence of smoking history. Current smokers were excluded from the search criteria. The CPRD EHR database was interrogated by CPRD researchers using a search engine enabled by the defined codelists and algorithms to create a pre-screened list of patients that met the protocol inclusion and exclusion criteria and were registered within practices close to the research sites in central London.

GP practices that had agreed to participate in research through CPRD were provided with a pre-screened list from which to identify and select suitable patients to receive information about the study. GPs gave confirmation to CPRD of patients who they thought were suitable for the study and received participant information packs from CPRD to send to potential recruits. The information pack contained a cover letter from the GP introducing the study, a patient information leaflet, and a response form that participants could complete and send to the research team in a pre-paid envelope to be contacted or to decline to take part. The research coordinator was then able to contact the participants, discuss the study, and invite them to enrol through a clinic appointment. Participants were required to carry a personal air monitor for 1 day a week for 6 months, undertake three lung function tests, record on a dairy card any exacerbations, sleep disturbance, changes to their regular treatment and to record their peak flow reading each morning.^3^ Output from the monitors were then linked with the EHR data to obtain information on COPD management, severity, co-morbidities and exacerbations. Linked data (Hospital Episode Statistics, Office of National Statistics, and Index of Multiple Deprivation/Townsend Score) was collected for enrolled patients. This study aimed to recruit 160 COPD patients. The study was reviewed by the Independent Scientific Advisory Committee (ref 15052) and approval confirmed by Camden and Islington Research Ethics Committee (ref 14/LO/2216). The CPRD database has been used for many epidemiological studies where patient data is accessed and used in publication. Annual research ethics approval from a NHS Health Research Authority (HRA) Research Ethics Committee (REC) is required to permit the collection and supply of anonymised patient data for Observational Research.

### Data availability

All data generated and analysed during the current study are available from the corresponding author on reasonable request.
